# Supplementary material for: Quantum illumination reveals phase-shift inducing cloaking
Source: Sci Rep. 2017 Aug 24;7:9333. doi: 10.1038/s41598-017-08505-w (PMC5571013; doi:10.1038/s41598-017-08505-w)
Supplement: Supplementary file 1 — Supplementary Information [file 41598_2017_8505_MOESM1_ESM.pdf]

SUPPLEMENTAL MATERIAL of
Quantum illumination reveals phase-shift inducing cloaking

U. Las Heras,[1] R. Di Candia,[1,2] K. G. Fedorov,[3,4] F. Deppe,[3,4,5] M. Sanz,[1] and E. Solano[1,6]

[1]*Department of Physical Chemistry, University of the Basque Country UPV/EHU, Apartado 644, E-48080 Bilbao, Spain*
[2]*Dahlem Center for Complex Quantum Systems, Freie Universität Berlin, 14195 Berlin, Germany*
[3]*Walther-Meissner-Institut, Bayerische Akademie der Wissenschaften, D-85748 Garching, Germany*
[4]*Physik-Department, Technische Universität München, D-85748 Garching, Germany*
[5]*Nanosystems Initiative Munich (NIM), Schellingstrae 4, 80799 München, Germany*
[6]*IKERBASQUE, Basque Foundation for Science, Maria Diaz de Haro 3, 48011 Bilbao, Spain*

In this Supplemental Material, we discuss details useful for the understanding of the main results of the paper.

Calculations

Here, we write down the protocol and compare the signal-to-noise ratios (SNR) of a quantum and the optimal classical protocols for detecting a cloaked object modelled as a dephaser introducing a phase ϕ in the bosonic field. For this, we consider a previous interaction with the environment that is modeled with a high-reflectivity beam splitter of reflectivity η. See the Fig. 1.

First, let us calculate the SNR of the classical protocol. Considering an initial coherent state $|\alpha\rangle$ with α real, we measure the quadrature in the same phase as the coherent state's (x since α is real), after interacting with the environment modeled as thermal noise, and then being dephased.

$$\begin{aligned}
\langle x''\rangle &= \langle (e^{-i\phi}a' + e^{i\phi}a'^{\dagger})/\sqrt{2}\rangle = \langle (e^{-i\phi}(\sqrt{\eta}a + \sqrt{1-\eta}a_h) + e^{i\phi}(\sqrt{\eta}a^{\dagger} + \sqrt{1-\eta}a_h^{\dagger}))\rangle/\sqrt{2} \\
&= \sqrt{\eta/2}(e^{-i\phi} + e^{i\phi})\alpha = \sqrt{2\eta}\cos(\phi)\alpha \qquad (1)\\
\langle x''^2\rangle &= \langle (e^{-i\phi}a' + e^{i\phi}a'^{\dagger})^2/2\rangle = \frac{\eta}{2}\langle (e^{-i2\phi}a^2 + e^{i2\phi}a^{\dagger 2} + 2a^{\dagger}a + 1)\rangle + \frac{1-\eta}{2}\langle (2a_h^{\dagger}a_h + 1)\rangle \\
&= \eta(\alpha^2 2\cos^2\phi + 1/2) + (1-\eta)(n_{th} + 1/2) \qquad (2)\\
\left(\frac{S^2}{\sigma^2}\right)_C &= \frac{\langle \bar{x}''\rangle^2}{\langle \bar{x}''^2\rangle - \langle \bar{x}''\rangle^2} = \frac{2\eta N(1-\cos(\phi))^2}{\eta/2 + (1-\eta)(n_{th} + 1/2)} \qquad (3)
\end{aligned}$$

where $N = \langle\alpha|a^{\dagger}a|\alpha\rangle$ is the number of photons sent in the signal beam, and $\bar{x} = \langle x\rangle|_{\phi=0} - \langle x\rangle$ in such a way the SNR is zero when $\phi = 0$. Notice that $\langle \bar{x}''^2\rangle - \langle \bar{x}''\rangle^2 = \langle x''^2\rangle - \langle x''\rangle^2$.

A. Quantum protocol with quadrature measurements

Let us do the same employing now a two-mode squeezed state $|\Psi_{12}\rangle = \sqrt{a-\lambda^2}\sum_{n=0}^{\infty}\lambda^n|n,n\rangle$. In the protocol, the signal beam interacts with the environment as in the classical protocol and then it is dephased, while the idler beam stays in the lab. When the signal beam reaches the lab the measurement $(x_1x_2 - p_1p_2)$ is performed.

$$\begin{aligned}
\langle x_1''x_2'' - p_1''p_2''\rangle &= \langle (e^{-i\phi}a' + e^{i\phi}a'^{\dagger})(a_2 + a_2^{\dagger}) - (1/i)^2(e^{-i\phi}a' - e^{i\phi}a'^{\dagger})(a_2 - a_2^{\dagger})\rangle/2 = \\
&= \langle (e^{-i\phi}(\sqrt{\eta}a_1 + \sqrt{1-\eta}a_h) + e^{i\phi}(\sqrt{\eta}a_1 + \sqrt{1-\eta}a_h)^{\dagger})(a_2 + a_2^{\dagger}) \\
&+ (e^{-i\phi}(\sqrt{\eta}a_1 + \sqrt{1-\eta}a_h) - e^{i\phi}(\sqrt{\eta}a_1 + \sqrt{1-\eta}a_h)^{\dagger})(a_2 - a_2^{\dagger})\rangle/2 = \\
&= \langle \sqrt{\eta}(2e^{-i\phi}a_1a_2 + 2e^{i\phi}a_1^{\dagger}a_2^{\dagger})\rangle/2 = 2\sqrt{\eta}\cos\phi\frac{\lambda}{1-\lambda^2} = 2\sqrt{\eta}\cos\phi\sqrt{N(N+1)} \qquad (4)
\end{aligned}$$

$$
\begin{aligned}
\langle(x_1''x_2''-p_1''p_2'')^2\rangle &= \langle(x_1''x_2'')^2\rangle+\langle(p_1''p_2'')^2\rangle-\langle x_1''p_1''x_2''p_2''\rangle-\langle p_1''x_1''p_2''x_2''\rangle\\
&= \frac{1}{4}[\langle\Big((\eta(e^{-i2\phi}a_1^2+e^{i2\phi}a_1^{\dagger 2}+a_1^\dagger a_1+a_1a_1^\dagger)+(1-\eta)(a_h^\dagger a_h+a_ha_h^\dagger)\Big)(a_2^2+a_2^{\dagger 2}+a_2^\dagger a_2+a_2a_2^\dagger)\rangle\\
&+ \langle\Big((\eta(e^{-i2\phi}a_1^2+e^{i2\phi}a_1^{\dagger 2}-a_1^\dagger a_1-a_1a_1^\dagger)+(1-\eta)(-a_h^\dagger a_h-a_ha_h^\dagger)\Big)(a_2^2+a_2^{\dagger 2}-a_2^\dagger a_2-a_2a_2^\dagger)\rangle\\
&+ \langle\Big((\eta(e^{-i2\phi}a_1^2-e^{i2\phi}a_1^{\dagger 2}+a_1^\dagger a_1-a_1a_1^\dagger)+(1-\eta)(a_h^\dagger a_h-a_ha_h^\dagger)\Big)(a_2^2-a_2^{\dagger 2}+a_2^\dagger a_2-a_2a_2^\dagger)\rangle\\
&+ \langle\Big((\eta(e^{-i2\phi}a_1^2-e^{i2\phi}a_1^{\dagger 2}-a_1^\dagger a_1+a_1a_1^\dagger)+(1-\eta)(-a_h^\dagger a_h+a_ha_h^\dagger)\Big)(a_2^2-a_2^{\dagger 2}-a_2^\dagger a_2+a_2a_2^\dagger)\rangle\\
&= \frac{1}{4}\langle[4\eta(e^{-i2\phi}a_1^2a_2^2+e^{i2\phi}a_1^{\dagger 2}a_2^{\dagger 2}+2a_1^\dagger a_1a_2^\dagger a_2+a_1^\dagger a_1+a_2^\dagger a_2+1)\\
&+ (1-\eta)(2a_h^\dagger a_ha_2^\dagger a_2+a_h^\dagger a_h+a_2^\dagger a_2+1)]]\rangle\\
&= \eta(1+8N(N+1)\cos^2\phi)+(1-\eta)(2n_{th}N+n_{th}+N+1)
\end{aligned}
\tag{5}
$$

Putting everything together, we have the SNR of the quantum protocol is given by

$$
\left(\frac{S^2}{\sigma^2}\right)_Q=\frac{\langle\bar{x}_1''\bar{x}_2''-\bar{p}_1''\bar{p}_2''\rangle^2}{\langle(\bar{x}_1''\bar{x}_2''-\bar{p}_1''\bar{p}_2'')^2\rangle-\langle\bar{x}_1''\bar{x}_2''-\bar{p}_1''\bar{p}_2''\rangle^2}=\frac{4\eta N(N+1)(1-\cos(\phi))^2}{\eta(1+4N(N+1)\cos^2\phi)+(1-\eta)(2n_{th}N+n_{th}+N+1)}
\tag{6}
$$

being $n_th=\mathrm{Tr}(\rho_{th}a_h^\dagger a_h)$ the average number of photons in the thermal environment and $N=\langle\Psi_{12}|a_1^\dagger a_1|\Psi_{12}\rangle=\langle\Psi_{12}|a_2^\dagger a_2|\Psi_{12}\rangle$ the average number of photons in each beam of the two-mode squeezed state. Equivalently to the notation used above, in the classical protocol, $\langle x_1''x_2''-p_1''p_2''\rangle=\langle x_1''x_2''-p_1''p_2''\rangle|_{\phi=0}-\langle x_1''x_2''-p_1''p_2''\rangle$.

In order to improve the classical protocol the condition $\left(\frac{S^2}{\sigma^2}\right)_Q>\left(\frac{S^2}{\sigma^2}\right)_C$ should hold,

$$
\frac{\left(\frac{S^2}{\sigma^2}\right)_Q}{\left(\frac{S^2}{\sigma^2}\right)_C}=\frac{(N+1)(1+\frac{1-\eta}{\eta}(2n_{th}+1))}{1+4N(N+1)\cos^2\phi+\frac{1-\eta}{\eta}(2n_{th}N+n_{th}+N+1)}>1
\tag{7}
$$

Firstly, let us point out that, in the limit $N\gg 1$ there is no gain, so we will focus on the limit $N\ll 1$. Considering the worst scenario in which $\cos^2\phi=1$, we get the expression

$$
\begin{gathered}
(N+1)(1+\tfrac{1-\eta}{\eta}(2n_{th}+1))-1-4N(N+1)-\tfrac{1-\eta}{\eta}(2n_{th}N+n_{th}+N+1)>0,\\
4N^2+3N-\tfrac{1-\eta}{\eta}n_{th}<0.
\end{gathered}
\tag{8}
$$

Since the equation has to be negative and the parabola has a positive second derivative, the solutions of the inequality are the ones between the roots of the equation with $N>0$.

$$
0<N<\frac{-3\pm\sqrt{9+16\frac{1-\eta}{\eta}n_{th}}}{8},
\tag{9}
$$

Therefore, there exists always a finite N enhancing the classical protocol a for any dephase ϕ only if $\frac{1-\eta}{\eta}n_{th}>0$.

Let us study now the different regimes for a small number of photons N in the two-mode squeezed state. The ratio of the quantum and classical SNR can be approximated as:

$$
\frac{\left(\frac{S^2}{\sigma^2}\right)_Q}{\left(\frac{S^2}{\sigma^2}\right)_C}\approx\frac{1+\frac{1-\eta}{\eta}+2\frac{1-\eta}{\eta}n_{th}}{1+\frac{1-\eta}{\eta}+\frac{1-\eta}{\eta}n_{th}}-\frac{(3+\frac{1-\eta}{\eta}n_{th})(1+\frac{1-\eta}{\eta}+2\frac{1-\eta}{\eta}n_{th})N}{(1+\frac{1-\eta}{\eta}+\frac{1-\eta}{\eta}n_{th})^2}.
\tag{10}
$$

Considering the most physical scenario in which $n_{th}\gg 1$ is high due to the noisy environment and a highly-reflective background $\frac{1-\eta}{\eta}$, there are three possible regimes parametrized by the product $\frac{1-\eta}{\eta}n_{th}$. The first case, in which $\frac{1-\eta}{\eta}n_{th}\gg 1$, the gain is 2, as one may observe from Eq. (10). On the other hand, when $\frac{1-\eta}{\eta}n_{th}\ll 1$, there is no gain, as discussed above. For intermediate regimes, we may observe that the zero-order term in Eq. (10) grows monotonously in $\frac{1-\eta}{\eta}n_{th}$, so one obtains a halfway gain.

B. Quantum protocol with Josephson mixer and photodetection

Here, we calculate the SNR of the protocol including a Josephson mixer (JM) and measuring the number of photons both in the signal and idler beams. Then, we rest them taking into account the gain introduced by the JM in order

to vanish the term proportional to $a_1''^\dagger a_1''$, which cannot be neglected for high n_{th}. In the following, we calculate the different factors.

$$a_2^{(3)\dagger}a_2^{(3)} = Ga_2''^\dagger a_2'' + (G-1)a_1''^\dagger a_1'' + \sqrt{G(G-1)}(a_1''a_2'' + a_1''^\dagger a_2''^\dagger), \quad (11)$$

$$a_1^{(3)\dagger}a_1^{(3)} = (G-1)a_2''a_2''^\dagger + Ga_1a_1''^\dagger + \sqrt{G(G-1)}(a_1''a_2'' + a_1''^\dagger a_2''^\dagger). \quad (12)$$

By measuring the photon number in both beams, we can compute results of the operator $O = Ga_2^{(3)\dagger}a_2^{(3)} - (G-1)a_1^{(3)\dagger}a_1^{(3)}$,

$$\begin{aligned}
\langle O\rangle &= \langle (G-1) + (2G-1)a_2''^\dagger a_2'' + \sqrt{G(G-1)}(a_1''a_2'' + a_1''^\dagger a_2''^\dagger)\rangle \\
&= (G-1) + (2G-1)N + 2\sqrt{G(G-1)}\eta\cos(\phi)\sqrt{N(N+1)}, \quad (13)\\
\langle O^2\rangle &= \langle (2G-1)^2 a_2''^\dagger a_2'' + (G-1)^2 + G(G-1)(a_1''a_2'' + a_1''^\dagger a_2''^\dagger)^2 + 2(G-1)[(2G-1)a_2''^\dagger a_2'' \\
&+ \sqrt{G(G-1)}(a_1''a_2'' + a_1''^\dagger a_2''^\dagger)] + (2G-1)\sqrt{G(G-1)}\{a_2''^\dagger a_2'', a_1''a_2'' + a_1''^\dagger a_2''^\dagger\}\rangle \\
&+ (2G-1)\sqrt{G(G-1)}\{a_2''^\dagger a_2'', a_1''a_2'' + a_1''^\dagger a_2''^\dagger\} \\
&= (2G-1)^2(2N^2+N) + (G-1)^2 + G(G-1)[4\eta N)(N+1)\cos(2\phi) + \eta(2N^2+1) \\
&+ (1-\eta)(1+N+n_{th}+2Nn_{th})] + 2(G-1)[(2G-1)N + \sqrt{G(G-1)}2\eta\sqrt{N(N+1)}\cos\phi] \\
&+ (2G-1)\sqrt{G(G-1)}2\sqrt{\eta}\cos(\phi)\sqrt{N(N+1)}(4N+1). \quad (14)
\end{aligned}$$

Now, we are able to obtain the SNR, which can be written as follows:

$$\begin{aligned}
\left(\frac{S^2}{\sigma^2}\right)_Q &= \frac{\langle\bar{O}\rangle^2}{\langle\bar{O}^2\rangle - \langle\bar{O}\rangle^2} \quad (15)\\
S^2 &= 4\eta G(G-1)N(N+1)(1-\cos(\phi))^2 \quad (16)\\
\sigma^2 &= 1 + N + N^2 + 2n_{th} + 2N\eta - 2n_{th}\eta - G(3 + 4N^2 + 5n_{th} - 5n_{th}\eta + N(7 + 2n_{th} + 3\eta - 2n_{th}\eta)) \\
&+ G^2(2 + 4N^2 + 3n_{th} - 3n_{th}\eta + N(7 + 2n_{th} + \eta - 2n_{th}\eta)) \\
&+ 2\sqrt{G(G-1)}\sqrt{N(N+1)}((2G-1)(1+2N) + 2(G-1)\sqrt{\eta})\sqrt{\eta}\cos(\phi) + 4G(G-1)M(M+1)\eta\cos(\phi)^2. \quad (17)
\end{aligned}$$

We now study the ratio with respect to the classical protocol in the limit of $N \to 0$:

$$\frac{\left(\frac{S^2}{\sigma^2}\right)_Q}{\left(\frac{S^2}{\sigma^2}\right)_C} \approx \frac{1 + 2n_{th}(1-\eta)}{1 + n_{th}(1-\eta)} - \frac{2(2G-1)(1 + 2n_{th}(1-\eta))\sqrt{\eta}\cos(\phi)}{\sqrt{G(G-1)}(1 + n_{th}(1-\eta))^2}\sqrt{N}. \quad (18)$$

We consider a gain by the JM $G = 1 + \epsilon$ with $\epsilon \to 0$ and a high number of thermal photons coming from the reflective background $n_{th}(1-\eta) \gg 1$. In this approximation, the Eq.(25) yields,

$$\frac{\left(\frac{S^2}{\sigma^2}\right)_Q}{\left(\frac{S^2}{\sigma^2}\right)_C} \approx 2 - \left(4\sqrt{\frac{\eta N}{\epsilon}} + 1\right)\frac{1}{n_{th}(1-\eta)}. \quad (19)$$

Hence, if we choose $\epsilon \gtrsim M$, the gain over the classical protocol is optimal.

Additionally, we consider imperfect photocounters for the implementation. Their efficiency is defined by a beam splitter of reflectivity χ that entangles the incoming field with vacuum. Hence, the number operators after the beam splitter can be written as follows:

$$\begin{aligned}
a_i^{(4)\dagger}a_i^{(4)} &= (\sqrt{\chi}a_i^{(3)\dagger} + \sqrt{1-\chi}a_{hi}^\dagger)(\sqrt{\chi}a_i^{(3)} + \sqrt{1-\chi}a_{hi}) \\
&= \chi a_i^{(3)\dagger}a_i^{(3)} + (1-\chi)a_{hi}^\dagger a_{hi} + \sqrt{\chi(1-\chi)}(a_i^{(3)\dagger}a_{hi} + a_i^{(3)}a_{hi}^\dagger), \quad (20)
\end{aligned}$$

where a_i corresponds to the field in the signal (i=1) or idler (i=2) beams and a_{hi} is the field of the vacuum entangled with the corresponding channel. Then, the operator O yields:

$$\begin{aligned}
O &= Ga_2^{(4)\dagger}a_2^{(4)} - (G-1)a_1^{(4)\dagger}a_1^{(4)} = \chi(+(2G-1)a_2''^\dagger a_2'' + \sqrt{G(G-1)}(a_1''a_2'' + a_1''^\dagger a_2''^\dagger)) \\
&+ (1-\chi)(Ga_{h2}''^\dagger a_{h2}'' - (G-1)a_{h1}''^\dagger a_{h1}'') + \sqrt{\chi(1-\chi)}[G[(\sqrt{G}a_2''^\dagger + \sqrt{G-1}a_1'')a_{h2} + (\sqrt{G}a_2'' + \sqrt{G-1}a_1''^\dagger)a_{h2}^\dagger] \\
&- (G-1)[(\sqrt{G}a_1''^\dagger + \sqrt{G-1}a_2'')a_{h1} + (\sqrt{G}a_1'' + \sqrt{G-1}a_2''^\dagger)a_{h1}^\dagger]]. \quad (21)
\end{aligned}$$

The expectation value $\langle O\rangle$ is the same as in Eq. (13), since the terms proportional to $(1-\chi)$ and $\sqrt{\chi(1-\chi)}$ vanish for vacuum. Similarly, we compute the expectation value of O^2, where the unique terms that survive are:

$$
\begin{aligned}
\langle O^2\rangle &= \chi^2\langle[G(a_2^{(3)\dagger}a_2^{(3)})-(G-1)(a_1^{(3)\dagger}a_1^{(3)})]^2\rangle+\chi(1-\chi)\langle(a_i^{(3)\dagger}a_{hi}+a_i^{(3)}a_{hi}^{\dagger})^2\rangle\\
&= \chi^2\langle[(G-1)+(2G-1)a_2''^{\dagger}a_2''+\sqrt{G(G-1)}(a_1''a_2''+a_1''^{\dagger}a_2''^{\dagger})]^2\rangle\\
&+ \chi(1-\chi)\langle[G^2(Ga_2''^{\dagger}a_2''+(G-1)a_1''^{\dagger}a_1''+\sqrt{G(G-1)}(a_1''a_2''+a_1''^{\dagger}a_2''^{\dagger}))\\
&+ Ga_1''^{\dagger}a_1''+(G-1)a_2''^{\dagger}a_2''+\sqrt{G(G-1)}(a_1''a_2''+a_1''^{\dagger}a_2''^{\dagger})]\rangle\\
&= \chi^2\langle(2G-1)^2(2N^2+N)+(G-1)^2+G(G-1)[4\eta N)(N+1)\cos(2\phi)+\eta(2N^2+1)\\
&+ (1-\eta)(1+N+n_{th}+2Nn_{th})]+2(G-1)[(2G-1)N+\sqrt{G(G-1)}2\eta\sqrt{N(N+1)}\cos\phi]\\
&+ (2G-1)\sqrt{G(G-1)}2\sqrt{\eta}\cos(\phi)\sqrt{N(N+1)}(4N+1)\rangle+\\
&+ \chi(1-\chi)\langle[G^2(GN+(G-1)(1+\eta N+(1-\eta)n_{th})+\sqrt{G(G-1)}2\eta\cos(\phi)\sqrt{N(N+1)})\\
&+ (G-1)^2(G(\eta N+(1-\eta)n_{th})+(G-1)(N+1)+\sqrt{G(G-1)}2\eta\cos(\phi)\sqrt{N(N+1)})]\rangle. \qquad (22)
\end{aligned}
$$

Then, in the limit $N\to 0$ the SNR yields at first order:

$$
\begin{aligned}
\frac{\left(\frac{S^2}{\sigma^2}\right)_Q}{\left(\frac{S^2}{\sigma^2}\right)_C} &\approx \frac{\chi G(1+2n_{th}(1-\eta))}{(1-\chi)+2G^2(1+n_{th}(1-\eta)+G(-2+3\chi+(-1+2\chi)(1-\eta)))}\\
&- \frac{\chi G^2(1+2n_{th}(1-\eta))\sqrt{\eta}((G-1)^2+G^2)\sqrt{\eta}(1-\chi)-\chi+2G\chi)}{\sqrt{G(G-1)}[(1-\chi)+2G^2(1+n_{th}(1-\eta)+G(-2+3\chi+(-1+2\chi)(1-\eta)))]^2}. \qquad (23)
\end{aligned}
$$

Finally, considering the same approximations as in Eq. (19), that is, $G=1+\epsilon$ with $\epsilon\to 0$ and $n_{th}(1-\eta)\gg 1$ we obtain:

$$
\frac{\left(\frac{S^2}{\sigma^2}\right)_Q}{\left(\frac{S^2}{\sigma^2}\right)_C}\approx\frac{2\chi}{1+2\epsilon(1-\chi)}+\left(4\chi\sqrt{\frac{\eta N}{\epsilon}}+1\right)\frac{1}{n_{th}(1-\eta)}, \qquad (24)
$$

which in case of using perfect photocounters,$\chi=1$, Eq. (19) is recovered. From here, one can compute the efficiency of the photocounters in order to get an improvement over the classical protocol:

$$
\chi>\left[2-\left(4\sqrt{\frac{\eta N}{\epsilon}}\right)\frac{1}{n_{th}(1-\eta)}\right]^{-1}\approx\frac{1}{2}+\left(2\sqrt{\frac{\eta N}{\epsilon}}\right)\frac{1}{n_{th}(1-\eta)}, \qquad (25)
$$

where the gain must fulfill $\epsilon=G-1>\eta N/(n_{th}(1-\eta))^2$.
